# Target Coverage Improvement With Dose Matching in Carbon-Ion Radiation Therapy for Pancreatic Cancer

**DOI:** 10.1016/j.ijpt.2025.101201

**Published:** 2025-08-29

**Authors:** Yohsuke Kusano, Hiroyuki Katoh, Yoshiki Takayama, Junya Nagata, Shogo Kurokawa, Terufumi Kusunoki, Koh Imura, Keisuke Tsuchida, Daisaku Yoshida, Tadashi Kamada, Atsushi Ito, Shinichi Minohara

**Affiliations:** 1 Section of Medical Physics and Engineering, Kanagawa Cancer Center, Yokohama, Japan; 2 Department of Radiation Oncology, Kanagawa Cancer Center, Yokohama, Japan

**Keywords:** Carbon-ion radiation therapy, Pancreatic cancer, Interfractional motion, Dose matching, Adaptive radiation therapy

## Abstract

**Purpose:**

Although carbon-ion radiation therapy (CIRT) creates a sharp dose distribution, inaccurate irradiation positioning may reduce the tumor dose. In pancreatic CIRT, interfractional tumor motion is a factor causing tumor dose reduction. This motion is typically accounted for in the planning target volume, and it cannot provide sufficient margin because the tumor is surrounded by the gastrointestinal tract. Online adaptive radiation therapy (ART) can solve this problem, but other problems such as equipment design and excessive time consumption remain in CIRT. The purpose of this feasibility study was to evaluate the effectiveness of dose matching (DM), which is more convenient than ART in pancreatic CIRT.

**Materials and Methods:**

On the in-room computed tomography images, search isocenters were placed 3 dimensionally around the isocenter determined by target matching (TM) at 0.2 cm intervals. The fractional dose distributions were then calculated at each isocenter. The coordinate with the best clinical target volume coverage (CTV V95%) was determined as the DM isocenter. In actual treatment, the use of couch shifting is assumed for irradiation in accordance with the DM isocenter. To evaluate the effectiveness of DM, variations from the initial plan for CTV V95% (ie, ΔCTV V95%) and organ-at-risk (OAR) dose (ΔD_OAR_) in bone matching (BM), TM, and DM were compared.

**Results:**

The median ΔCTV V95% values in BM, TM, and DM were −2.18%, −1.39%, and −0.36%, respectively. DM significantly improved CTV V95%. OAR doses were within their limits. Toxicity in DM was considered equivalent to that in BM because the maximum ΔD_OAR_ in DM was similar to the BM results.

**Conclusion:**

DM significantly improved CTV V95% in pancreatic CIRT within dose constraints of OARs. However, DM should be properly applied by considering treatment efficacy and efficiency. The appropriate use of TM, DM, and online/offline ART is required for each treatment site to improve the target coverage.

## Introduction

The Bragg peak of a carbon-ion beam enables the delivery of a highly concentrated radiation dose to a tumor while reducing the radiation dose to adjacent organs.^1^, ^2^, ^3^ However, it is sensitive to density variations along the beam path, and such variations can reduce tumor dose and increase the organ-at-risk (OAR) exposure.^4^, ^5^, ^6^, ^7^, ^8^, ^9^ In addition to density variations, including changes in the patient physique and the amount of gastrointestinal gas present, interfractional motion of the tumor and OARs are critical factors to be considered for accurate irradiation.^9^ Overall, these factors may reduce the tumor dose and increase the gastrointestinal tract dose.

Although image-guided radiation therapy with bone matching (BM) cannot account for interfractional motions, adaptive radiation therapy (ART) modifies the treatment plan before irradiation, and interfractional motions can be considered.^10^, ^11^, ^12^, ^13^, ^14^, ^15^ Recently, online ART using magnetic resonance imaging (MRI) has been introduced in photon radiation therapy.^16^, ^17^, ^18^ During this process, however, the patient is immobilized on the treatment couch for an extended period during replanning, causing discomfort to the patient and reducing the treatment throughput.^10^ MRI-guided online ART is still challenging in particle therapy compared with photon radiation therapy owing to many problems, such as the effect of magnetic fields on the beam, equipment design, and excessive time consumption.^19^ Li et al^20^ evaluated the efficacy of ART in passive carbon-ion radiation therapy (CIRT) for pancreatic cancer using in-room computed tomography (irCT) images. Target coverage was improved compared with BM and target matching (TM), but they stated that many issues, including software and hardware aspects, need to be resolved before clinical use. Jia et al^21^ evaluated the efficacy of ART in scanning CIRT for lung cancer. Although target coverage was improved, they stated that ART increases the workload of the facility and increases the time and effort required by clinicians and physicists. Thus, various problems need to be resolved for ART to be used in particle therapy. This study focused on target coverage improvement by couch shift using irCT images acquired on the treatment day. For target coverage improvement by couch shift, the position of irradiation is usually adjusted by marker matching (MM) by inserting metal into the target organ^22^, ^23^ or TM.^24^ However, this is not sufficient in some cases, and further technical improvement is needed.

In this feasibility study, to further improve dose delivery to the tumor, we investigated dose matching (DM), which allows to determine the best irradiation positioning. Similar studies have been conducted for lung, head-and-neck, and prostate cancers in proton radiation therapy,^25^, ^26^ but the efficacy of DM for pancreatic cancer and CIRT has not been evaluated. Further, no detailed comparative evaluation of multiple matching methods (eg, BM, TM, and DM) is available to the best of our knowledge. In CIRT, DM may effectively improve the target coverage until the persistent problems of ART are resolved.

## Methods

### Patient selection

Twenty consecutive patients who underwent scanning CIRT for pancreatic cancer at our hospital from January 2019 to September 2022 with at least 4 irCT scans acquired during the treatment period were selected (Table 1). During the treatment period, irCT images were acquired at our facility at least once per week considering the patient's physical condition, x-ray exposure, and treatment throughput. As irradiation was performed in 4 fractions per week over 3 weeks, the irCT scans were typically obtained 3 times throughout the treatment. For patients who underwent irCT scans at least 4 times, the frequency of confirmation was increased because their body conditions (eg, gastrointestinal gas positions) changed during the treatment period, causing a decrease in target coverage and high dose delivery to the gastrointestinal tract. In all selected patients, replanning was not performed during treatment. This single-center study was conducted according to the guidelines of the Declaration of Helsinki and approved by the Institutional Review Board (Approval no. 2019eki-106 granted on August 30, 2021). Informed consent was obtained from all the patients enrolled in the study, and their data were anonymized.

**Table 1:** Characteristics of patients with pancreatic cancer enrolled in this study.

Patient	Age (years)	Sex	UICC TNM (8th ed.)	Stage	Location	No. irCT scans
1	60	Male	cT4N0M0	3	H	6
2	79	Male	cT3N0M0	2A	H	7
3	54	Female	cT4N0M0	3	H	6
4	64	Male	cT3N1M0	2B	H&B	5
5	86	Female	cT3N0M0	2A	H	4
6	78	Female	cT1cN0M0	1A	H	4
7	75	Male	cT4N0M0	3	H	6
8	65	Female	cT4N0M0	3	H&B	4
9	72	Male	cT4N0M0	3	H	7
10	61	Male	cT4N1M0	3	H	4
11	79	Male	cT1bN0M0	1A	H	5
12	77	Female	cT4N0M0	3	H	4
13	59	Female	cT1cN0M0	1A	H	4
14	78	Female	cT4N0M0	3	B	4
15	75	Female	cT2N0M0	1B	B	4
16	84	Male	cT3N0M0	2A	H	6
17	74	Male	cT1cN0M0	1A	H	5
18	75	Female	cT4N0M0	3	B	6
19	79	Male	cT1cN0M0	1A	H	6
20	67	Male	cT4N0M0	3	B	5

**Abbreviations:** UICC, Union for International Cancer Control; H, pancreatic head; B, pancreatic body; H&B, pancreatic head and body.

### Initial plan

#### CT images

Two immobilization devices (Blue BAG BodyFix, Elekta; Shellfitter, Kuraray) were used for all patients. The patients fasted for at least 5 h before the initial planning CT (pCT) scan or treatment, and if the patient had not defecated within the previous 24 h, an enema was performed. Four-dimensional CT scans were performed using a pCT scanner (Aquilion LB, Canon Medical Systems). At our facility, the irradiation directions at gantry angles of 0°, 165°, and 270° are generally used for pancreatic CIRT based on previous studies (Supplementary Figure 1-1).^9^ Considering that the irradiation system is a fixed beam,^27^ each irradiation direction is adjusted based on the combination of beam nozzle angles (0° for vertical beam or 90° for horizontal beam), patient posture (supine or prone), and rotation and roll angle of the treatment couch. pCT images were acquired for each patient posture and roll angle of the treatment couch because the locations of organs changed. All patients enrolled in this study (Table 1) underwent pCT scanning in the supine (roll of 0°) and prone (roll of 15°) positions.

#### Contouring

The gross tumor volume (GTV) was delineated on pCT images at 10 respiratory time points using contouring software (MIM Maestro version 6.9.6 to 7.2.8, MIM Software), and then a phase range comprising movement within 0.5 cm was determined.^28^ The clinical target volume (CTV) was defined as the GTV with a 0.5 cm margin and included the local regional elective nodal and neuroplexus regions.^29^ In addition, the entire pancreas was included in the CTV as a preventive region, regardless of the tumor site. The CTV was delineated on all pCT images within the determined phase range, and the summed CTV was defined as the internal CTV. Similarly, the gastrointestinal tracts were delineated on all pCT images within the determined phase range, and the summed contour was defined as the planning risk volume (PRV). The spinal cord, kidney, and skin were delineated on the pCT images of the maximum exhalation phase (pCT_50%_ images). The planning target volume (PTV) was prepared with a 0.3 cm margin to the internal CTV, which was reduced when the PTV was near or overlapping with the PRV. Contouring was performed on both supine and prone CT images.

#### Dose distribution optimization

In pancreatic CIRT, the prescribed relative-biological-effectiveness-weighted absorbed dose per beam is set at 4.6 Gy.^29^ At our facility, the fractions in the total relative biological effectiveness-weighted dose of 55.2 Gy are typically 4, 6, and 2 at gantry angles of 0°, 165°, and 270°, respectively (Supplementary Figure 1-1).^9^, ^29^ Only 1 beam per day is irradiated to the patient. Irradiation at gantry angles of 0° and 270° is performed in the supine position, and that at a gantry angle of 165° is performed in the prone position.

**Figure 1:**
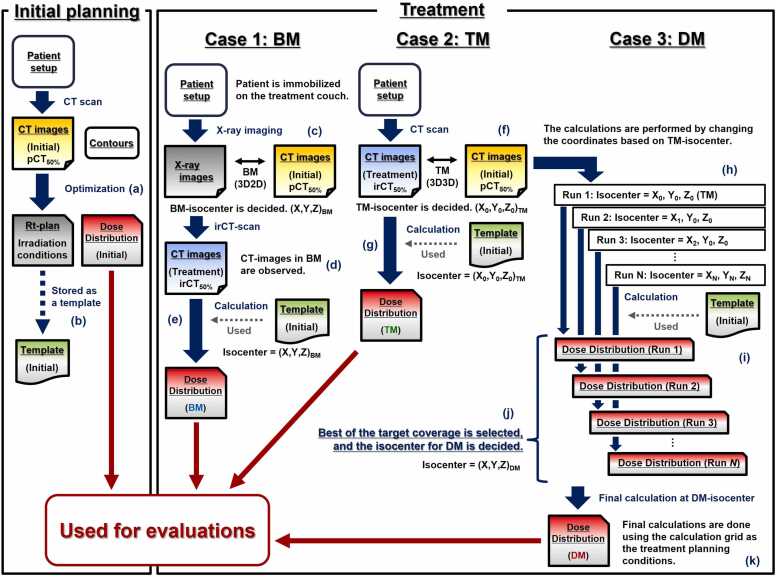
Evaluation workflows for BM, TM, and DM. (a) Initial plan dose distributions are optimized. (b) Optimized irradiation conditions are stored as templates. (c) BM: (d) Fractional CT images in BM are observed, and (e) fractional dose distributions are calculated based on the BM isocenter. (f) TM: (g) Fractional dose distributions are calculated based on the TM isocenter. (h) DM: Search isocenters are placed in a grid at 0.2-cm intervals in LR, SI, and AP directions, (i) fractional dose distributions in each search isocenter are calculated, (j) best target coverage is selected, and isocenter for DM is determined. (k) Fractional dose distributions in DM are calculated using the calculation grid as the treatment planning conditions.

In this study, 2 types of clinical CT images acquired in the supine and prone positions were used. The dose distributions were optimized using a treatment planning system (Monaco for Carbon version 6.1, Elekta). In supine CT images, in addition to 0° and 270° real beams, a virtual 165° beam in the prone position was optimized. In prone CT images, in addition to the real 165° beam, virtual beams of 0° and 270° in the supine position were optimized (Supplementary Figure 1-2). The total dose, dose per beam, and number of passes per beam followed common conditions from clinical practice. The mean-replacement method^9^ was adopted to prepare a robust initial plan for gastrointestinal gas. To comply with the specifications by the Japan Carbon-Ion Radiation Oncology Study Group (J-CROS),^29^ the dose constraint was targeted within 44 Gy at the maximum dose covering 2 cm^3^ (D_2 cm^3^_) of the gastrointestinal tract PRV. The dose constraint imposed by J-CROS was 46 Gy. The maximum doses covering 0.01 cm^3^ (D_max_) for the spinal cord and 0.5 cm^3^ (D_0.5 cm^3^_) for the skin were restricted to remain below 30 and 48 Gy, respectively. Under these conditions, the initial plan dose distributions were optimized such that 95% of the prescribed dose covered the PTV using pCT_50%_ images and contours (Figure 1a). To calculate the dose distributions for each treatment day, the optimized irradiation conditions were stored as templates (Figure 1b).

### In-room CT (irCT) scans

In each treatment, the patient position was adjusted by 2-/3-dimensional BM using front- and lateral-view x-ray and pCT_50%_ images (Figure 1c, Supplementary Figure 2-1). Following the adjustments, carbon-ion scanning beams were irradiated (CI-1000, TOSHIBA, Tokyo, Japan).^30^, ^31^ Fractional CT images, before or after irradiation, were acquired in the maximum exhalation phase (irCT_50%_ images) while maintaining the same patient setup as that during treatment (Figure 1d).^27^, ^32^ The GTV, CTV, and OARs were delineated on irCT_50%_ images.

### Calculation of fractional dose distributions

#### Bone matching

The fractional dose distributions of the 3 gantry angles for each treatment day were calculated based on the BM isocenter projected as a marker on the irCT_50%_ images while maintaining the irradiation conditions determined in the initial plan using templates (Figure 1e, Supplementary Figure 2-1).

#### Target matching

Considering an existing method,^24^ TM was performed by rigid registration of the MIM Maestro software with organs, including the pancreas, around the GTV as indices, and the position was manually adjusted as required (Figure 1f, Supplementary Figure 2-2). The pCT_50%_ and irCT_50%_ images were used for TM. Following adjustment, the isocenter at initial planning, which was shifted to match the result of TM, was transferred to the irCT_50%_ images (TM isocenter). The fractional dose distributions in TM were calculated based on the TM isocenter (Figure 1g).

#### Dose matching

In DM, the dose distributions in multiple isocenters were calculated based on the TM isocenter, and the isocenter with the best target coverage was determined (Supplementary Figure 2-3). The isocenters for search were placed in a grid at 0.2 cm intervals in the left-right (LR), superior-inferior (SI), and anterior-posterior (AP) directions (Figure 1h). Based on the coordinates of the TM isocenter, the placement range was set to ±0.4 cm along each direction. The axis in the beam direction was fixed to the TM isocenter. The dose distributions at those isocenters were then calculated using the scripting function of Monaco (Figure 1i). Following the calculations, the coordinate with the best CTV irradiated by more than 95% of the prescribed dose (CTV V95%) was determined as the DM isocenter (Figure 1j). Assuming clinical use, the initial plan CTV contour that was copied in the TM condition was used for analysis. In actual treatment, the displacement in the LR, SI, and AP directions from the coordinate under BM or TM should be determined based on the DM isocenter, and the treatment couch should be shifted to perform treatment irradiation.

As the calculations required fast processing for clinical use of DM, the calculation grid of the Monaco parameter was set to 0.6 cm in the dose distribution calculation at each search isocenter. The calculation grid is typically 0.2 cm. As this parameter increases, the resolution of the dose distribution decreases (Supplementary Figure 3). In this study, the calculation grid was set to 0.2 cm, and the fractional dose distribution at the DM isocenter was recalculated for the comparative evaluation of BM, TM, and DM. (Figure 1k).

### Evaluation of CTV coverage and OAR dose

CTV V95% for each treatment day (CTV V95%_irCT_) was analyzed with the total dose and evaluated CTV V95%_irCT_ variations (ΔCTV V95%) for each matching method from the initial plan using the following formula:

(1) $$∆\mathrm{CTV V}95\%={\mathrm{CTV V}95\%}_{\mathrm{irCT}}-{\mathrm{CTV V}95\%}_{\mathrm{pCT}},$$

where CTV V95%_pCT_ is CTV V_95%_ in the initial plan. For the OAR doses, the variation in D_2 cm^3^_ for the stomach, duodenum, colon, small intestine, and kidney, in addition to D_max_ for the spinal cord and D_0.5 cm^3^_ for skin from the initial plan were evaluated as follows:

(2) $${∆D}_{\mathrm{OAR}}=D_{\mathrm{OAR},\mathrm{irCT}}-D_{\mathrm{OAR},\mathrm{pCT}},$$

where ΔD_OAR_ represents the variation in the OAR dose from the initial plan, D_OAR,irCT_ is the OAR dose on each treatment day for each matching method, and D_OAR,pCT_ is the OAR dose in the initial plan. As described in Section of CT images, CT images in the supine and prone positions were used in this study. Therefore, an additional analysis of both supine and prone positions was performed.

### Analysis of interfractional tumor motion

The interfractional tumor motion was analyzed based on the center of gravity of the CTV delineated on pCT_50%_ (initial planning) and irCT_50%_ (treatment with BM) images. To match the center coordinates of both CT images, the CTV center of gravity coordinates were corrected based on coordinate of each isocenter. Subsequently, the displacements in the LR, SI, and AP directions (ΔPos(LR, SI, AP)_CTV_) were obtained by taking the difference from the initial plan with reference to the isocenter as follows:

(3) $${∆\mathrm{Pos}\left(\mathrm{LR},\mathrm{SI},\mathrm{AP}\right)}_{\mathrm{CTV}}=\left({\mathrm{XYZ}}_{\mathrm{CTV},\mathrm{irCT}}-{\mathrm{XYZ}}_{\mathrm{ISO},\mathrm{irCT}}\right)-\left({\mathrm{XYZ}}_{\mathrm{CTV},\mathrm{pCT}}-{\mathrm{XYZ}}_{\mathrm{ISO},\mathrm{pCT}}\right),$$

where XYZ_CTV,irCT_ and XYZ_CTV,pCT_ represent the CTV center of gravity coordinates on the irCT and pCT images, respectively, and XYZ_ISO,irCT_ and XYZ_ISO,pCT_ represent the isocenters on the irCT and pCT images, respectively. The analyses were performed for the 3 patterns of all positionings, as well as supine and prone positions.

### Statistical analysis

ΔCTV V95% and ΔD_OAR_ between the 3 matching methods were evaluated using CT images from the same patient. The Friedman test, suitable for non-normally distributed data, was employed to compare significant differences between the 3 groups. As the comparison of the 3 groups was evaluated 3 times, the obtained *P* values were multiplied by 3 using the Bonferroni method. Finally, the statistical tests were conducted considering *P* < .05 as the significance level. The SPSS statistical software (IBM SPSS Statistics, version 29.0, IBM) was used for analysis.

In the evaluation of the interfractional tumor motion, the Wilcoxon signed-rank test was used to assess the 2 groups of supine and prone positions for the LR, SI, and AP directions owing to the absence of normality in each data set.

## Results

### Interfractional tumor motion

**Figure 2:**
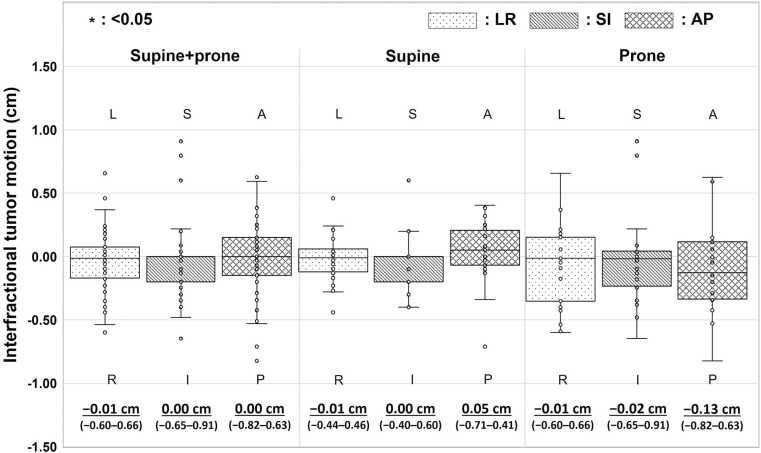
Interfractional tumor motion after BM. Boxplots showing median, first and third quartiles, maximum, minimum, and outliers for displacements from initial plan in LR, SI, and AP directions for each patient position (L, left; R, right; S, superior; I, inferior; A, anterior; P, posterior). The upper values indicate the median values, and the values in parentheses indicate the minimum-maximum values.

Figure 2 shows the boxplots of interfractional tumor motion. No significant differences were observed between the supine and prone positions; however, the prone position showed greater variability in all directions.

### Variations in CTV coverage

**Figure 3:**
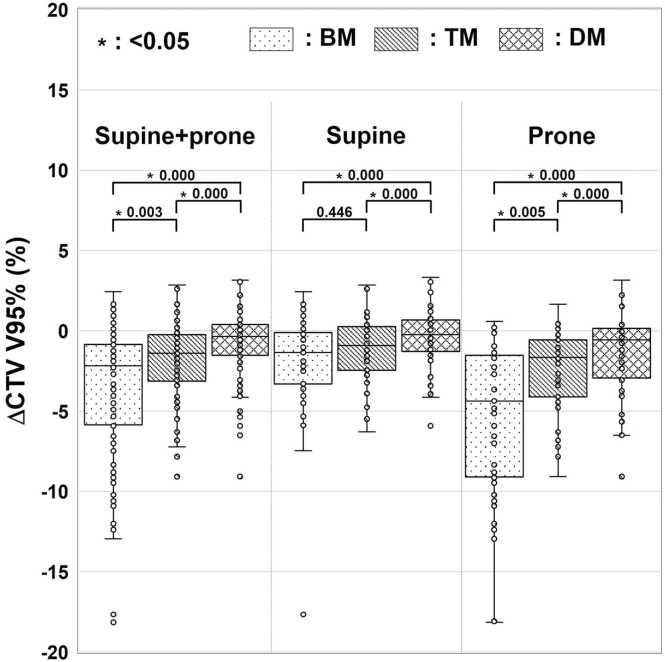
Evaluation results of variations in CTV coverage (ΔCTV V95%) from the initial plan. The median, first and third quartiles, maximum, minimum, and outliers for ΔCTV V95% in each matching method are presented in a boxplot.

Table 2a lists the absolute values of CTV V95%. The median CTV V95% was the highest for DM, followed by TM and BM. Figure 3 shows the ΔCTV V95% values for BM, TM, and DM. The prone position was more variable than the supine position regarding CTV V95%. Significant differences were observed, except for BM-TM in the supine position, and CTV V95% was the best for DM, followed by TM and BM.

**Table 2:** CTV coverage and OAR doses.

a) CTV coverage											
Patient positioning		DVH.P		CTV V95%_pCT_ (%)		CTV V95%_irCT_ (%)					
						BM		TM		DM	
Supine + Prone		V95%		97.86 (93.67-99.92)		95.85 (76.63-99.91)		96.71 (90.26-99.93)		97.54 (90.78-99.95)	
Supine		V95%		97.84 (94.02-99.92)		95.92 (76.63-99.91)		96.71 (90.420-99.93)		97.42 (90.78-99.95)	
Prone		V95%		98.11 (93.67-99.90)		93.47 (81.36-99.42)		96.67 (90.26-99.56)		97.78 (92.09-99.89)	

b) OAR doses in all positions											
Organ	Contour type		DVH.P		D_OAR,pCT_ (Gy)		D_OAR,irCT_ (Gy)				
							BM		TM		DM
Small intestine	p50%		D_2 cm^3^_		7.16 (0.94-36.43)		10.00 (0.74-46.43)		11.96 (0.80-46.09)		13.96 (0.87-46.86)
	PRV		D_2 cm^3^_		9.11 (1.02-36.68)		—		—		—
Stomach	p50%		D_2 cm^3^_		29.46 (11.50-43.41)		30.16 (3.24-46.37)		33.52 (6.13-46.26)		34.18 (1.70-45.91)
	PRV		D_2 cm^3^_		34.94 (14.95-44.80)		—		—		—
Duodenum	p50%		D_2 cm^3^_		29.95 (16.79-40.97)		36.19 (9.87-46.93)		38.74 (12.61-46.73)		38.52 (11.14-46.79)
	PRV		D_2 cm^3^_		34.16 (24.73-42.78)		—		—		—
Colon	p50%		D_2 cm^3^_		5.87 (4.19-34.91)		13.58 (4.26-46.96)		10.49 (4.26-38.69)		11.37 (4.49-41.05)
	PRV		D_2 cm^3^_		7.64 (4.19-36.51)		—		—		—
Spinal cord	p50%		D_0.01 cm^3^_		16.27 (15.45-17.96)		16.40 (15.33-18.28)		16.40 (15.42-18.78)		16.38 (15.44-18.62)
Right kidney	p50%		D_2 cm^3^_		7.33 (2.97-10.12)		7.00 (2.30-36.88)		7.00 (2.66-24.07)		6.77 (3.03-27.08)
Left kidney	p50%		D_2 cm^3^_		19.60 (12.30-34.33)		19.86 (2.36-40.41)		19.94 (2.68-42.98)		19.83 (1.40-40.57)
Skin	p50%		D_0.5 cm^3^_		15.29 (13.59-16.49)		15.33 (13.68-16.54)		15.35 (13.66-16.53)		15.35 (13.64-16.52)

*Note:* Top values indicate median values, and values in parentheses indicate minimum-maximum values.

**Abbreviations:** CTV, clinical target volume; V95%, volume irradiated by at least 95% of the prescribed dose; DVH.P, dose-volume histogram parameter; CTV V95%_pCT_, CTV coverage in initial plan; CTV V95%_irCT_, CTV coverage on each treatment day; BM, bone matching; TM, target matching; DM, dose matching; OAR, organ at risk; D_OAR,pCT_, OAR dose in initial plan; D_OAR,irCT_, OAR dose on each treatment day; Gy, relative-biological-effectiveness-weighted absorbed dose; p50%, contour delineated on maximum exhalation phase; PRV, planning risk volume; D_2 cm^3^_, maximum dose covering 2.0 cm^3^; D_0.01 cm^3^_, maximum dose covering 0.01 cm^3^; D_0.5 cm^3^_, maximum dose covering 0.5 cm^3^.

### Variations in OAR dose

**Figure 4:**
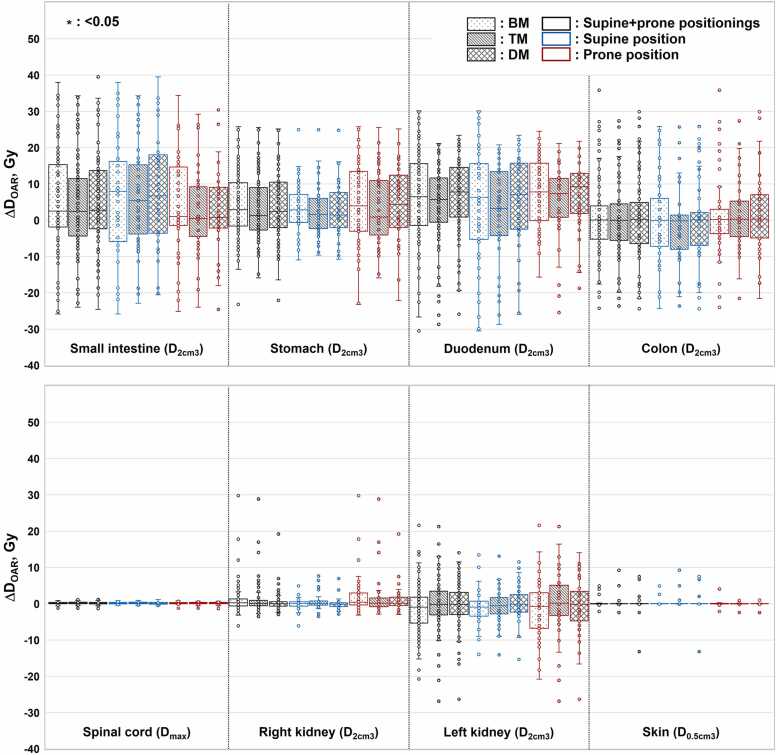
Evaluation results of variations from the initial plan in ΔD_OAR_. The median, first, and third quartiles, maximum, minimum, and outliers for ΔD_OAR_ for each matching method are presented in a boxplot.

The maximum gastrointestinal tract dose was within 1 Gy, but it exceeded the dose constraint required by J-CROS (Table 2b). Nevertheless, the value was acceptable when evaluated for all fractions. Values for the spinal cord and skin were within the dose constraints. Figure 4 shows ΔD_OAR_ for BM, TM, and DM. No significant differences were observed between the supine and prone positions.

### Calculation times

The fusion and workflow functions of the MIM Maestro software and the script function of Monaco were used in DM. By adjusting the calculation grid, the dose distribution calculation during the isocenter search could be reduced to <20 s per calculation point. However, up to 90 min were required per beam to complete the entire process, hindering use in clinical settings. This can be attributed to the time required for script processing and the use of different systems for the sequence of DM processes. If DM can be implemented in a single software package, the calculation time may be suitable for clinical use.

## Discussion

In this study, the effectiveness of DM was evaluated to improve target coverage. To evaluate the effectiveness of DM, both BM and TM were also assessed for comparison. The results showed improved target coverage in TM and DM compared with BM, possibly because TM and DM corrected for interfractional tumor motion. Subsequently, a comparison of TM and DM showed that target coverage was further improved by DM. TM determines the isocenter that corrects only for the effect of changes in tumor position, whereas DM can determine the isocenter to achieve the best overall target coverage by including additional factors, such as gastrointestinal gas. DM is considered to be more effective than TM in improving target coverage.

A trend in interfractional tumor motion similar to that reported by Kubota et al^24^ was observed. One possible reason for the change in tumor position was the effect of patient setup. At our facility, every patient was immobilized on the treatment couch in a natural position, and the fitting of the shell was adjusted such that it was equivalent to that of the pCT scan. However, it was difficult to match these positions perfectly, and it was likely that the tumor position changed. As it was weighted on the abdomen, the prone position likely caused more changes in the organ position than the supine position, which was weighted on the dorsum. In addition, the respiratory change seemed to cause an added effect in the SI direction. Some patients tended to breathe out less at the time of treatment than during acquisition of the pCT scan. Consequently, the tumor position shifted to the inferior side in the long axis. The shift in the tumor position to the superior side in the long axis may be caused by stronger expiration compared with the shift in the pCT scan owing to breathlessness or nervousness. Another factor could be the effect of increasing or decreasing gastrointestinal gas. It would be extremely difficult to correct such changes in the tumor position by patient positioning or breath control.

According to a previous study, the variation in target coverage shown in Figure 3 includes the effects of changes in patient physique, gastrointestinal gas, and tumor position changes.^9^ Although the impact of changes in patient physique is greater when the weight increases or decreases, such cases were not included in this study. In the variation in target coverage, the effect of gastrointestinal gas was included because it was not completely removed, although its effect was mitigated by the mean-replacement method.^9^ Although several effects were included, the comparison between the changes in tumor position shown in Figure 2 and target coverage variations of BM shown in Figure 3 suggests that changes in tumor position affect the variation in target coverage. This result is consistent with a previous study.^24^ The decrease in target coverage was especially large in the prone position, which induced large changes in the tumor position. To improve target coverage with CIRT for pancreatic cancer, changes in the tumor position should be corrected in addition to the effect of gastrointestinal gas.

In terms of the OAR dose, no difference was found between the matching methods for the spinal cord, kidney, or skin. A higher dose in the left kidney compared with the right kidney was caused by the beam direction (Supplementary Figure 1-2). In clinical practice, the beam direction should be selected based on kidney function. In all matching methods, D_2 cm^3^_ for gastrointestinal tracts such as the small intestine, stomach, duodenum, and colon tended to be higher during treatment than during initial planning. In addition, these variations were larger than those of the spinal cord and skin, which experienced less organ movement; this could be attributed to the shifting position of gastrointestinal tracts.^22^ On the contrary, no significant differences were observed in the dose-volume histogram parameters between BM, TM, and DM, especially for their highest values, which were almost equal (Figure 4). Currently, pancreatic cancer is primarily treated at our institution using BM. Based on the analysis of dose-volume histogram parameters, the frequency of adverse events is expected to be similar to that under conventional BM, even if DM is adopted.

DM may be effective in treatment sites with high likelihood of interfractional tumor motion and insufficient PTV margins, in which the irradiation area is surrounded by the gastrointestinal tract. In this study, the efficacy of DM was evaluated in pancreatic cancer, and its effectiveness was demonstrated. Although DM is convenient for pancreatic CIRT, considering the treatment efficacy and efficiency, it is not suitable for all treatment sites. First, it is likely unsuitable for treatment sites where treatment planning is based on gradations in dose distribution because the isocenter is determined by prioritizing target coverage in DM. For example, in prostate cancer, the dose distribution near the rectum is low to reduce rectal damage.^33^, ^34^ If using DM, the rectal dose increases because the isocenter is determined by prioritizing target coverage. Although this may be improved by including the rectal dose condition in the determination of the DM isocenter, TM or MM may be sufficient considering the time required. Second, DM requires more time for dose distribution calculation, consequently reducing the treatment throughput. Furthermore, the patient is immobilized on the treatment couch for an extended period during calculation, possibly causing discomfort. Therefore, depending on the treatment site, treatment should be based on BM, MM, or TM. For example, in liver cancer, MM^35^ is considered adequate, as almost no OARs are close to the irradiation area, and the PTV margins, including interfractional tumor motion, can be set. In esophageal cancer, BM with robust planning for the intra/interfractional tumor or OAR motion and setting an appropriate PTV margin is considered adequate.^36^ Third, DM cannot respond to tumor growth. For example, in head-and-neck cancer and bone and soft-tissue tumors, because tumor growth or shrinkage is likely a problem,^37^, ^38^ online or offline ART (replanning) is likely effective. Overall, to improve target coverage, the selection of TM, DM, online or offline ART, and a robust planning technique should be conducted for each treatment site.

A limitation of this study is that only 20 patients were included, and the number of irCT scans ranged from 4 to 7 per patient during all treatments. Although an increase in the number of patients and irCT scans may cause changes in ΔCTV V95%, the sample size for examination is adequate and may not affect the reported DM effectiveness. Nevertheless, the analysis accuracy can be further improved. Although irCT scans were not performed on all treatment days, changes during the treatment period were likely reflected in the results of this study because the irCT images were acquired at least once per week. Second, to implement DM in clinical practice, irCT scans should be easy to perform. As a CT scanner cannot be installed at the irradiation position due to interference with the irradiation equipment, the CT scanner is placed at a distance from the irradiation position. Consequently, CT scanning is performed at an off-center position (Supplementary Figure 4). As a result, irCT scans are time-consuming and reduce the treatment throughput. At our facility, we currently perform TM only when necessary, and DM is assumed to follow the same policy. Improving irCT scans to facilitate their acquisition remains a major challenge in particle therapy.

## Conclusions

This study demonstrated that DM can significantly improve target coverage compared with BM and TM in scanning CIRT for pancreatic cancer. DM has the potential to improve clinical outcomes. Even when DM was used, variations in target coverage were observed in some cases. Therefore, when the method is implemented in clinical practice, it is necessary to prepare a treatment plan robust to various factors that affect the reduction of target coverage. In particle radiation therapy, the routine evaluation of dose distribution using irCT images throughout the treatment and appropriate actions are essential to improve the treatment outcomes.

## Funding

Dr Hiroyuki Katoh, Dr Daisaku Yoshida, and Dr Yohsuke Kusano received research funding from Toshiba Energy Systems and Solutions Corporation.

## Author Contributions

Yohsuke Kusano: Conceptualization, Methodology, Validation, Formal analysis, Investigation, Data curation, Writing- Original draft preparation, Visualization, Project administration, Funding acquisition. Hiroyuki Katoh: Writing- Original draft preparation, Writing–Review and Editing, Project administration, Funding acquisition. Yoshiki Takayama: Methodology, Validation, Writing- Review and Editing, Visualization. Junya Nagata: Validation, Data curation, Writing- Review and Editing. Shogo Kurokawa: Writing- Review and Editing. Terufumi Kusunoki: Writing- Review and Editing. Koh Imura: Writing- Review and Editing. Keisuke Tsuchida: Writing- Review and Editing. Daisaku Yoshida: Writing- Review and Editing, Funding Acquisition. Tadashi Kamada: Writing- Review and Editing. Atsushi Ito: Writing- Review and Editing. Shinichi Minohara: Writing- Review and Editing, Project administration.

## Declaration of Conflicts of Interest

Tadashi Kamada, MD, is an Associate Editor of the *International Journal of Particle Therapy*. The authors have no additional conflicts of interest to declare*.*

## Data Availability

Research data are stored at an institutional repository and can be shared upon request to the corresponding author.

## Footnotes

Appendix A Supplementary material associated with this article can be found in the online version at doi:10.1016/j.ijpt.2025.101201.
