# Supplementary material for: Target Coverage Improvement With Dose Matching in Carbon-Ion Radiation Therapy for Pancreatic Cancer
Source: Int J Part Ther. 2025 Aug 29;18:101201. doi: 10.1016/j.ijpt.2025.101201 (PMC12746089; doi:10.1016/j.ijpt.2025.101201)
Supplement: Supplementary file 1 — Supplementary material [file mmc1.docx]

**Supplementary Material #1**

**
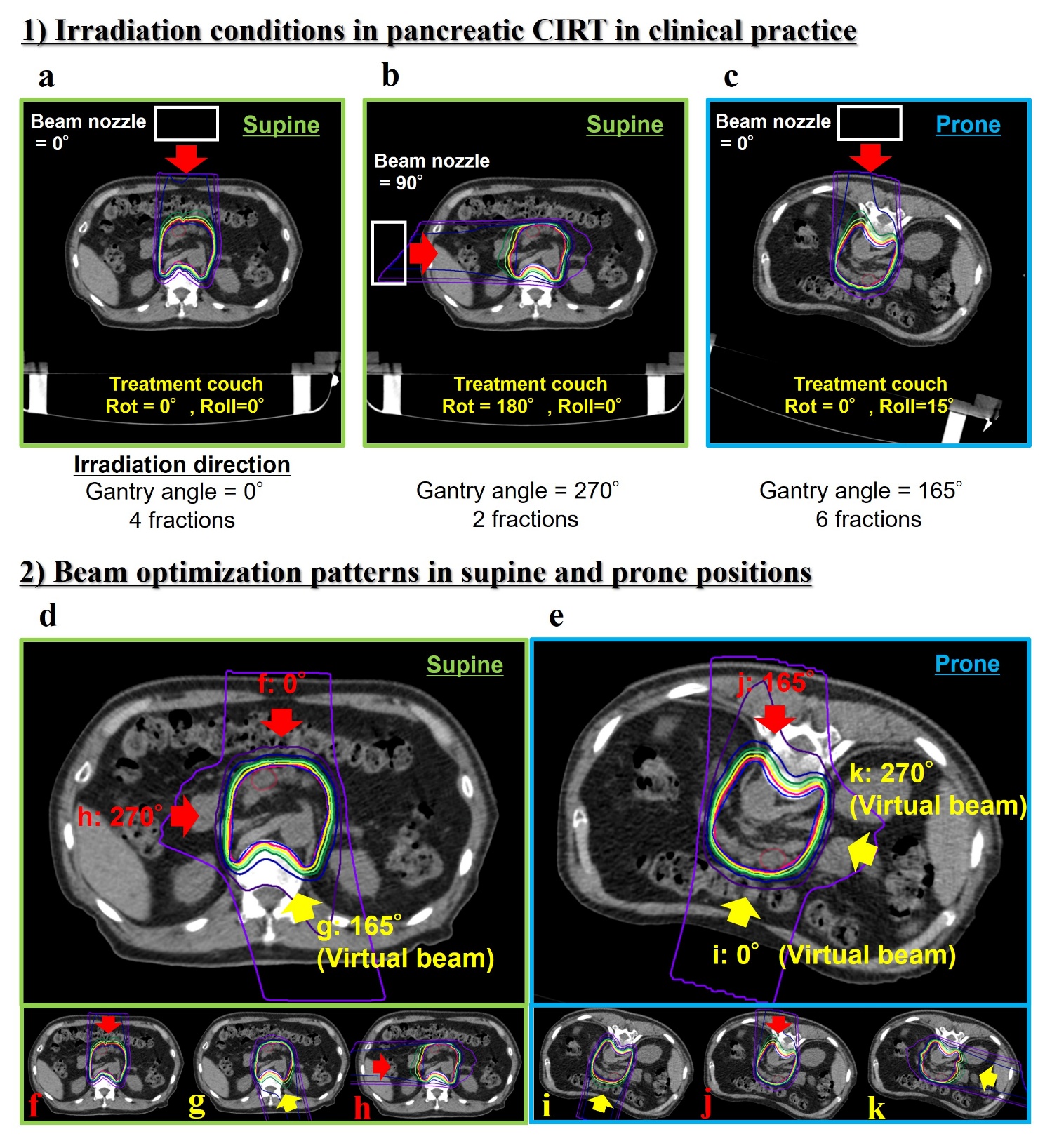
**

**Supplementary Figure 1.** Irradiation directions in clinical practice for pancreatic CIRT and beam optimization patterns used in this study.
