# Supplementary material for: Target Coverage Improvement With Dose Matching in Carbon-Ion Radiation Therapy for Pancreatic Cancer
Source: Int J Part Ther. 2025 Aug 29;18:101201. doi: 10.1016/j.ijpt.2025.101201 (PMC12746089; doi:10.1016/j.ijpt.2025.101201)
Supplement: Supplementary file 2 — Supplementary material [file mmc2.docx]

**Supplementary Material #2**

**
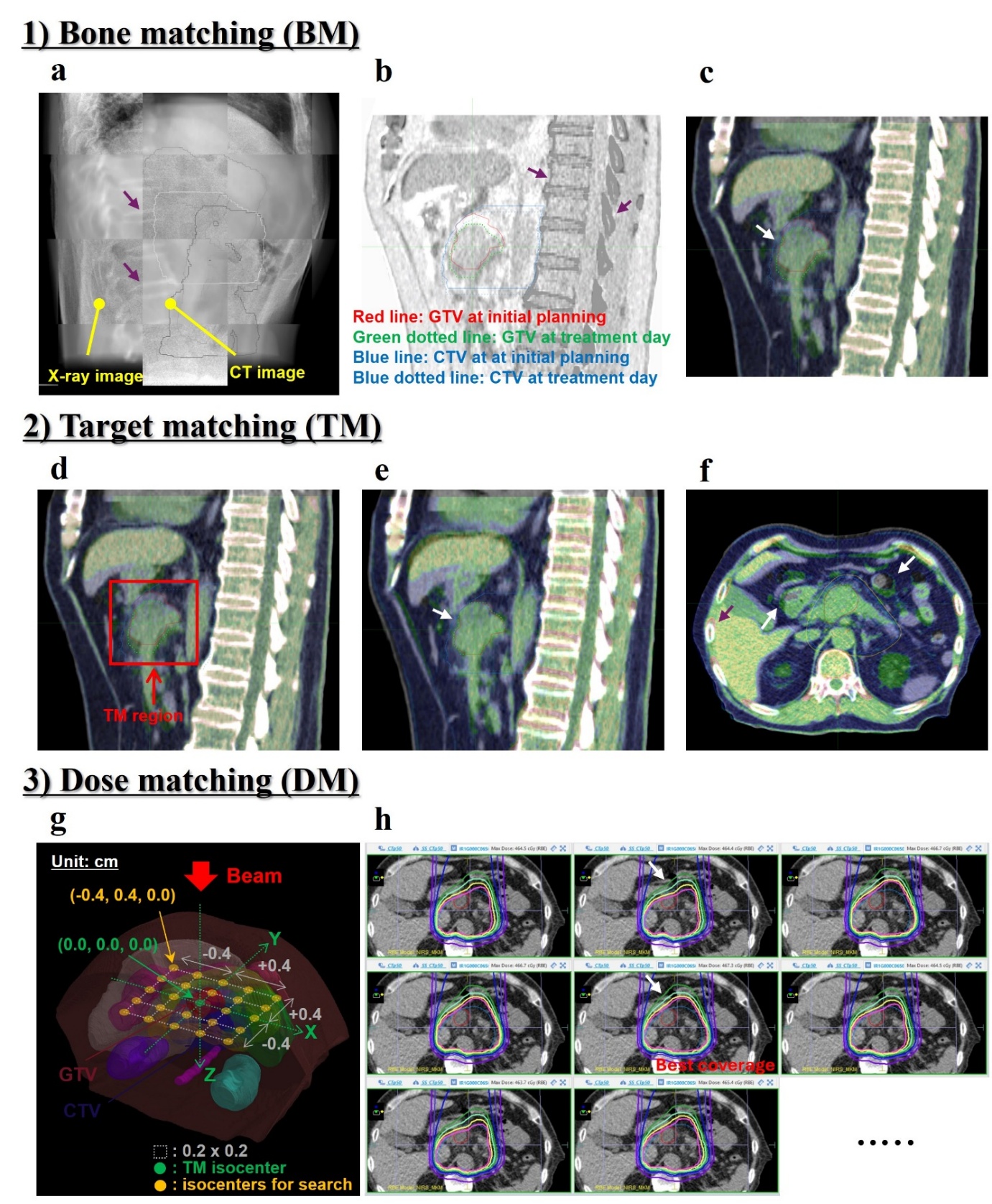
**

**Supplementary Figure 2.** Additional explanation of patient positioning methods for BM, TM, and DM. (2-1) Bone matching (BM). (a) Patient position adjusted by two-/three-dimensional BM using front- and lateral-view X-ray and initial planning computed tomography (CT) images of maximum exhalation phase (pCT_50%_ images). (b) Subtraction image after BM between pCT_50%_ images and fractional CT images of maximum exhalation phase (irCT_50%_ images). The bones are well matched. (c) Displaced tumor position. Regions in gray and green indicate pCT_50%_ and irCT_50%_ images, respectively. (2-2) Target matching (TM). (d) Region to perform TM. Region including GTVs of pCT50% and irCT50% images. (e) Suitably matched GTVs of pCT50% and irCT50% images after TM. (f) Displaced bone positions after TM. Positional changes in the gastrointestinal tract and variations in the position and volume of gastrointestinal gases occur across days. (2-3) Dose matching (DM). (g) Schematic diagram of DM. (h) Calculated dose distributions at each search isocenter to determine the isocenter with the best target coverage.
