# Supplementary figures and images for: Target Coverage Improvement With Dose Matching in Carbon-Ion Radiation Therapy for Pancreatic Cancer
Source: Int J Part Ther. 2025 Aug 29;18:101201. doi: 10.1016/j.ijpt.2025.101201 (PMC12746089; doi:10.1016/j.ijpt.2025.101201)

**Supplementary Material #4**

**
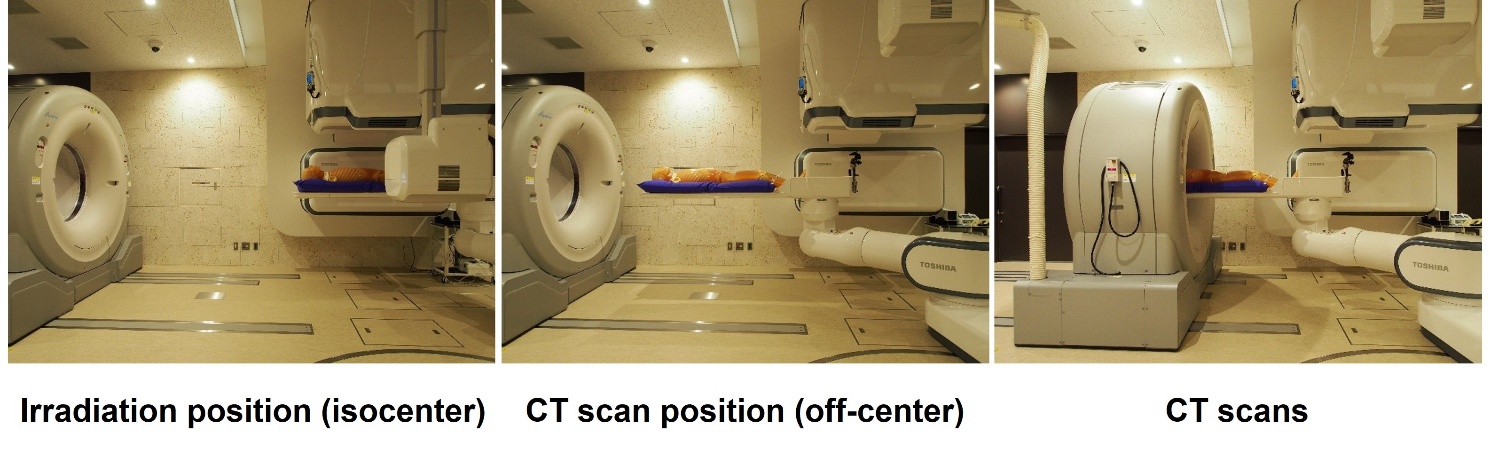
**

**Supplementary Figure 4.** Photographs of irCT scans.

Supplement: Supplementary file 4 — Supplementary material [file mmc4.docx]
